# Supplementary material for: Prior cueing affects the saccadic response to targets in the praying mantis Sphodromantis lineola
Source: J Exp Biol. 2025 Jun 11;228(11):jeb249296. doi: 10.1242/jeb.249296 (PMC12188249; doi:10.1242/jeb.249296)
Supplement: Supplementary information [file jexbio-228-249296-s1.pdf]

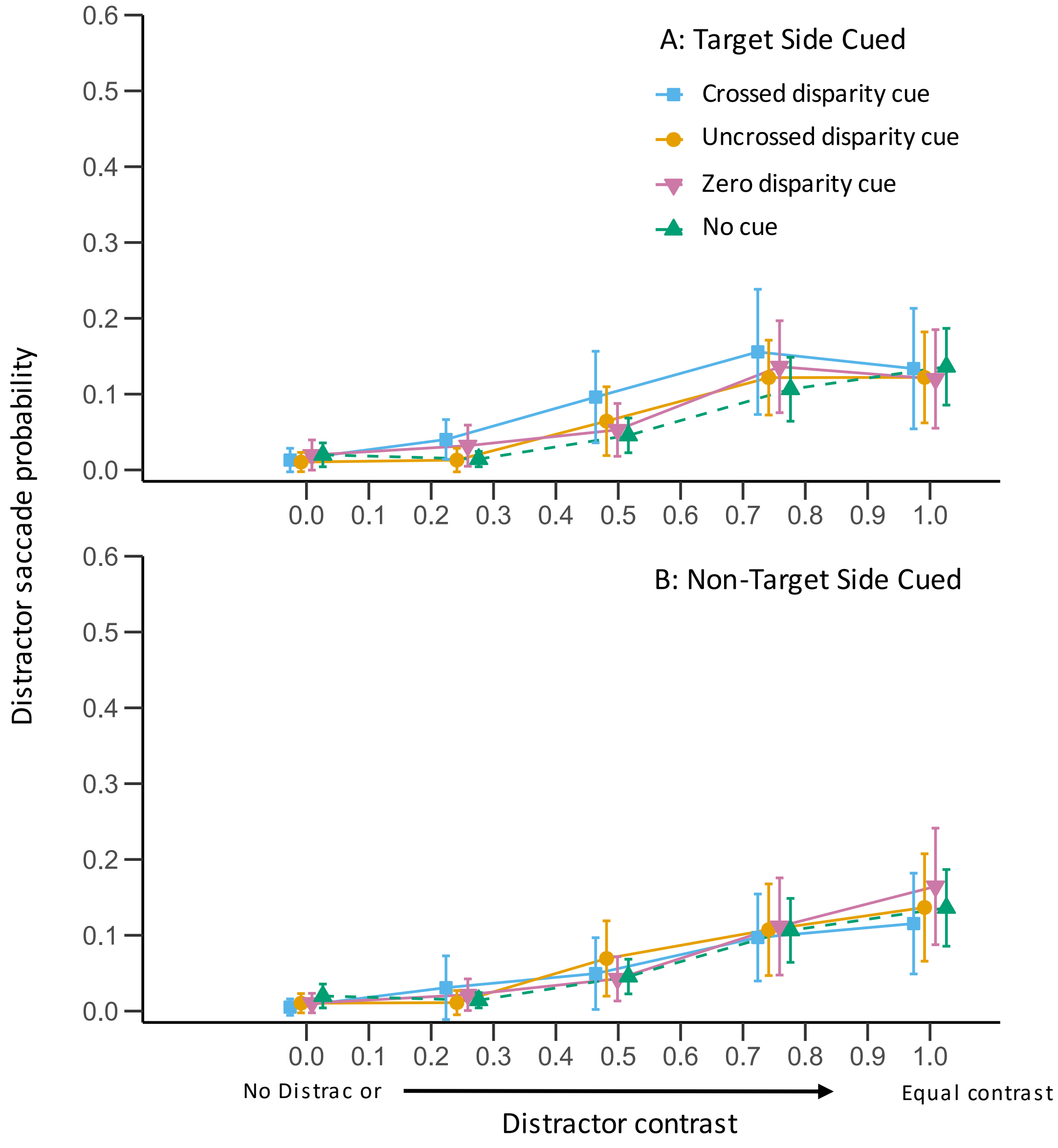

Fig. S1. The influence of cue position and disparity on distractor saccade probability. Mean (± 95% C.I.) proportion of trials with a first saccade towards the distractor as a function of its Michelson contrast (N=24 mantises). The cue was presented either on the side of the target (A) or on the side of the distractor (B). Blue curves represent trials with crossed disparity cues, yellow curves are for trials with uncrossed disparity cues while pink cues are for trial conducted with zero disparity cues. Green curves represent uncued trials and are the same across the two panels. The presented data combines the data from both 4 s (Experiment 1) and 100 ms (Experiment 2) cues. Overall, only the contrast of the distractor had a significant effect on the probability of first saccades towards the distractor (Model with contrast compared to null model: χ^2=334.75, df=1, p<0.001 ; Estimate ± standard error of the contrast=2.440±0.145, Z=16.79, p<0.001).

Journal of Experimental Biology • Supplementary information

Table S1. Summary of effects of distractor contrast and cuing condition with the Uncued condition as baseline. A) Estimates, standard errors, Wald test results and p-values of factors in the best model for the analysis of experiment 1 and 2. **B)** Bootstrapped 95% confidence intervals of the estimates presented in table A).

A)	Estimate	Std Error	Z Value	P-value	
Intercept - Uncued	-0.454	0.307	-1.482	0.138	
Cuing Condition - Crossed Disparity Target Cued	-0.268	0.157	-1.710	0.087	
Cuing Condition - Crossed Disparity Distractor Cued	-0.434	0.157	-2.768	0.006	**
Cuing Condition - Uncrossed Disparity Target Cued	-0.189	0.156	-1.213	0.225	
Cuing Condition - Uncrossed Disparity Distractor Cued	-0.442	0.156	-2.834	0.005	**
Cuing Condition - Zero Disparity Target Cued	-0.189	0.156	-1.211	0.226	
Cuing Condition - Zero Disparity Distractor Cued	-0.195	0.156	-1.251	0.211	
Contrast - Uncued	-2.601	0.177	-14.708	<0.001	***
Contrast : Cuing Condition - Crossed Disparity Target Cued	-0.006	0.315	-0.018	0.985	
Contrast : Cuing Condition - Crossed Disparity Distractor Cued	0.887	0.295	3.008	0.003	**
Contrast : Cuing Condition - Uncrossed Disparity Target Cued	0.359	0.300	1.197	0.231	
Contrast : Cuing Condition - Uncrossed Disparity Distractor Cued	1.112	0.288	3.855	<0.001	***
Contrast : Cuing Condition - Zero Disparity Target Cued	0.334	0.302	1.106	0.269	
Contrast : Cuing Condition - Zero Disparity Distractor Cued	0.389	0.300	1.300	0.194	

B)	Estimates	2.5%	97.5%
Intercept - Uncued	-0.454	-1.052	0.119
Cuing Condition - Crossed Disparity Target Cued	-0.268	-0.591	0.019
Cuing Condition - Crossed Disparity Distractor Cued	-0.434	-0.769	-0.129
Cuing Condition - Uncrossed Disparity Target Cued	-0.189	-0.480	0.095
Cuing Condition - Uncrossed Disparity Distractor Cued	-0.442	-0.761	-0.140
Cuing Condition - Zero Disparity Target Cued	-0.189	-0.501	0.084
Cuing Condition - Zero Disparity Distractor Cued	-0.195	-0.509	0.114
Contrast - Uncued	-2.601	-2.978	-2.266
Contrast : Cuing Condition - Crossed Disparity Target Cued	-0.006	-0.638	0.605
Contrast : Cuing Condition - Crossed Disparity Distractor Cued	0.887	0.265	1.487
Contrast : Cuing Condition - Uncrossed Disparity Target Cued	0.359	-0.240	0.966
Contrast : Cuing Condition - Uncrossed Disparity Distractor Cued	1.112	0.525	1.742
Contrast : Cuing Condition - Zero Disparity Target Cued	0.334	-0.244	0.937
Contrast : Cuing Condition - Zero Disparity Distractor Cued	0.389	-0.207	0.974

Journal of Experimental Biology • Supplementary information

Table S2. Summary of effects of distractor contrast and cuing condition with the Zero Disparity, Distractor Cued condition as baseline. A) Estimates, standard errors, Wald test results and p-values of factors in the best model for the analysis of experiment 1 and 2**. B)** Bootstrapped 95% confidence intervals of the estimates presented in table A).

A)	Estimate	Std Error	Z Value	P-value	
Intercept - Zero Disparity Distractor Cued	-0.650	0.320	-2.029	0.043	*
Cuing Condition - Crossed Disparity Target Cued	-0.073	0.181	-0.402	0.688	
Cuing Condition - Crossed Disparity Distractor Cued	-0.239	0.182	-1.318	0.188	
Cuing Condition - Uncrossed Disparity Target Cued	0.007	0.180	0.036	0.971	
Cuing Condition - Uncrossed Disparity Distractor Cued	-0.247	0.181	-1.366	0.172	
Cuing Condition - Zero Disparity Target Cued	0.006	0.181	0.032	0.975	
Cuing Condition - Uncued	0.195	0.156	1.253	0.210	
Contrast - Zero Disparity Distractor Cued	-2.212	0.244	-9.074	<0.001	***
Contrast : Cuing Condition - Crossed Disparity Target Cued	-0.395	0.357	-1.106	0.269	
Contrast : Cuing Condition - Crossed Disparity Distractor Cued	0.498	0.339	1.467	0.142	
Contrast : Cuing Condition - Uncrossed Disparity Target Cued	-0.031	0.343	-0.089	0.929	
Contrast : Cuing Condition - Uncrossed Disparity Distractor Cued	0.722	0.334	2.165	0.030	*
Contrast : Cuing Condition - Zero Disparity Target Cued	-0.055	0.346	-0.160	0.873	
Contrast : Cuing Condition - Uncued	-0.389	0.299	-1.302	0.193	

B)	Estimates	2.5%	97.5%
Intercept - Zero Disparity Distractor Cued	-0.650	-1.262	-0.041
Cuing Condition - Crossed Disparity Target Cued	-0.073	-0.400	0.268
Cuing Condition - Crossed Disparity Distractor Cued	-0.239	-0.588	0.130
Cuing Condition - Uncrossed Disparity Target Cued	0.007	-0.364	0.384
Cuing Condition - Uncrossed Disparity Distractor Cued	-0.247	-0.599	0.116
Cuing Condition - Zero Disparity Target Cued	0.006	-0.385	0.368
Cuing Condition - Uncued	0.195	-0.094	0.523
Contrast - Zero Disparity Distractor Cued	-2.212	-2.674	-1.744
Contrast : Cuing Condition - Crossed Disparity Target Cued	-0.395	-1.153	0.296
Contrast : Cuing Condition - Crossed Disparity Distractor Cued	0.498	-0.173	1.146
Contrast : Cuing Condition - Uncrossed Disparity Target Cued	-0.031	-0.776	0.656
Contrast : Cuing Condition - Uncrossed Disparity Distractor Cued	0.722	0.091	1.353
Contrast : Cuing Condition - Zero Disparity Target Cued	-0.055	-0.805	0.629
Contrast : Cuing Condition - Uncued	-0.389	-0.955	0.189

Journal of Experimental Biology • Supplementary information

Table S3. Summary of the effects of the cuing conditions compared to the Uncued condition for trials without a distractor.
A) Estimates, standard errors, Wald test results and p-values for each factors in the selected model**. B)** Bootstrapped 95% confidence intervals of the estimates presented in table A).

**A)**	**Estimate**	**Std Error**	**Z Value**	**P-value**	
Intercept - Uncued	-0.149	0.376	-0.396	0.692	
Cuing Condition - Crossed Disparity Target Cued	-0.199	0.195	-1.019	0.308	
Cuing Condition - Crossed Disparity Distractor Cued	-0.575	0.200	-2.869	0.004	**
Cuing Condition - Uncrossed Disparity Target Cued	-0.362	0.198	-1.830	0.067	
Cuing Condition - Uncrossed Disparity Distractor Cued	-0.556	0.200	-2.775	0.006	**
Cuing Condition - Zero Disparity Target Cued	-0.069	0.197	-0.350	0.726	
Cuing Condition - Zero Disparity Distractor Cued	-0.257	0.198	-1.297	0.195	

**B)**	**Estimates**	**2.5%**	**97.5%**
Intercept - Uncued	-0.149	-0.867	0.645
Cuing Condition - Crossed Disparity Target Cued	-0.199	-0.548	0.180
Cuing Condition - Crossed Disparity Distractor Cued	-0.575	-0.996	-0.179
Cuing Condition - Uncrossed Disparity Target Cued	-0.362	-0.776	-0.006
Cuing Condition - Uncrossed Disparity Distractor Cued	-0.556	-0.959	-0.116
Cuing Condition - Zero Disparity Target Cued	-0.069	-0.487	0.304
Cuing Condition - Zero Disparity Distractor Cued	-0.257	-0.633	0.148

Journal of Experimental Biology • Supplementary information

Table S4. Summary of effects of the cuing conditions compared to the Zero Disparity, Distractor cued condition for trials without a distractor. A) Estimates, standard errors, Wald test results and p-values of factors in the selected model**. B)** Bootstrapped 95% confidence intervals of the estimates presented in table A).

A)	Estimate	Std Error	Z Value	P-value
Intercept - Zero Disparity Distractor Cued	-0.405	0.394	-1.029	0.303
Cuing Condition - Crossed Disparity Target Cued	0.058	0.227	0.256	0.798
Cuing Condition - Crossed Disparity Distractor Cued	-0.318	0.232	-1.375	0.169
Cuing Condition - Uncrossed Disparity Target Cued	-0.106	0.230	-0.460	0.646
Cuing Condition - Uncrossed Disparity Distractor Cued	-0.299	0.231	-1.293	0.196
Cuing Condition - Zero Disparity Target Cued	0.188	0.229	0.818	0.413
Cuing Condition - Uncued	0.257	0.198	1.297	0.195

B)	Estimates	2.5%	97.5%
Intercept - Zero Disparity Distractor Cued	-0.405	-1.186	0.309
Cuing Condition - Crossed Disparity Target Cued	0.058	-0.376	0.506
Cuing Condition - Crossed Disparity Distractor Cued	-0.318	-0.797	0.153
Cuing Condition - Uncrossed Disparity Target Cued	-0.106	-0.562	0.386
Cuing Condition - Uncrossed Disparity Distractor Cued	-0.299	-0.765	0.218
Cuing Condition - Zero Disparity Target Cued	0.188	-0.310	0.644
Cuing Condition - Uncued	0.257	-0.132	0.657

Table S5. Summary of the model investigating the effect of cue position for trials where the distractor is absent. A) Estimates, standard errors, Wald test results and p-values of factors in the model**. B)** Bootstrapped 95% confidence intervals of the estimates presented in table A).

A)	Estimates	Std Error	Z Value	P-value	
Intercept	-0.307	0.379	-0.810	0.418	
Distractor Cued	-0.269	0.136	-1.975	0.048	*

B)	Estimates	2.5%	97.5%
Intercept	-0.307	-1.025	0.432
Distractor Cued	-0.269	-0.563	-0.0001

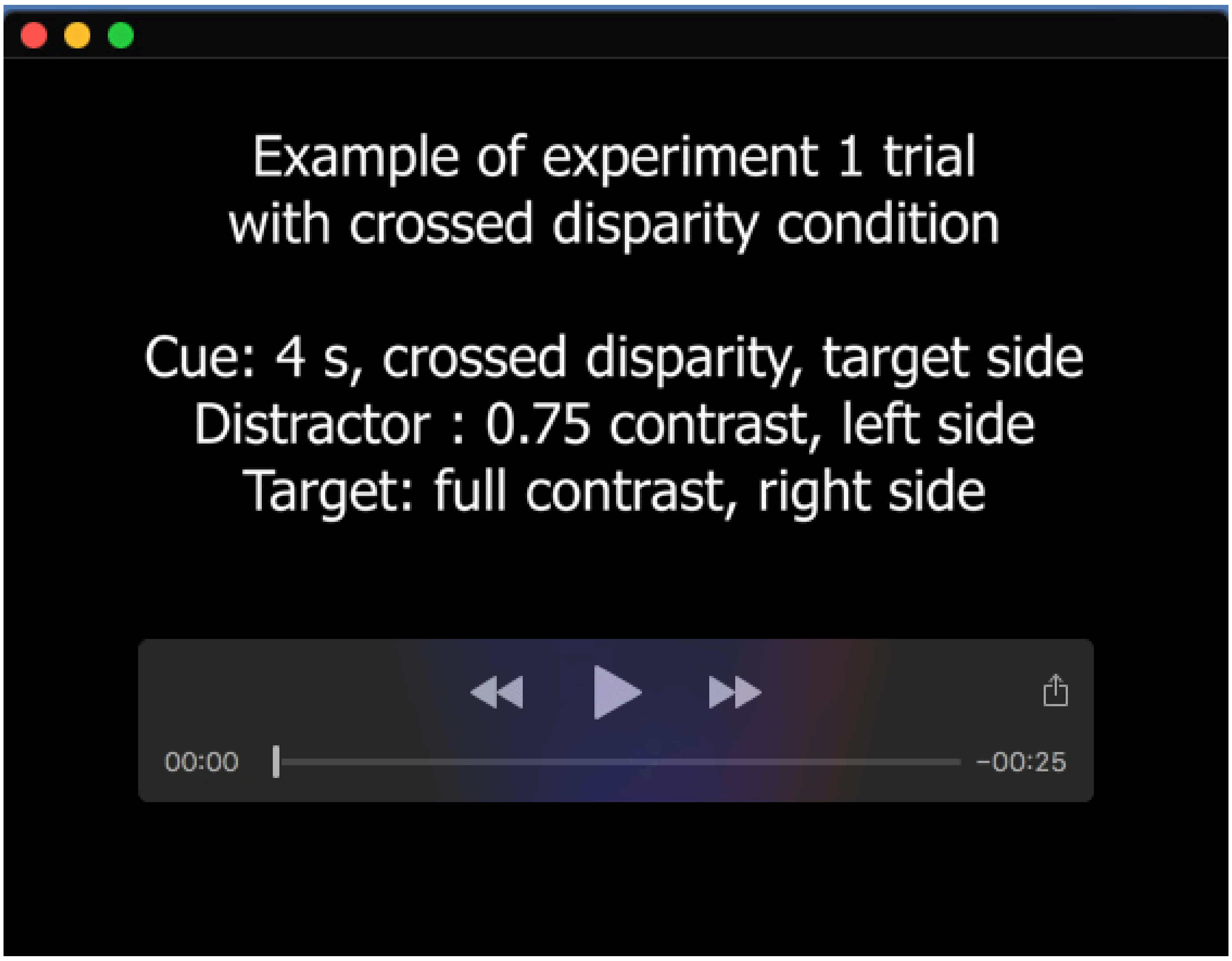

Movie 1. Examples of trials presented to mantises during the experiment 1 and example of mantis head saccade. This experiment used 4 s cues. The first trial shows a crossed disparity cue placed on the target side and a distractor with a 0.75 contrast. The second trial shows an uncrossed disparity cue, on the target side and a 0.49 contrast distractor. The third trial shows a Zero disparity cue on the side opposite to the target and a 0 contrast (invisible) distractor. Finally, the fourth trial shows an Uncued condition with a 0.25 distractor contrast. Each trial is preceded by the centring stimulus. These trials were shown to a mantis placed upside-down in front of the screen with a purple filter in front of its left eye and the red filter in front of its right eye. Please note that crossed and uncrossed disparity cues and the target and distractor each consist of two images (one perceived through the purple filter and the other through the red one). However, to balance perception of the two images through the two filters, the gain of the image for the purple filter was strongly reduced. Thus, this image may be difficult to perceive depending on the reader's screen settings. The last two videos are recordings of experimental trials made with the webcam placed at the foot of the experimental screen, filming the mantises from below. On the first video, the mantis performs head saccades to the left which corresponds to the right side of the screen. On the second video, the mantis performs saccades to the right, which corresponds to the left of the screen. At the bottom of the video there is a mirror reflecting a small part of the screen (hidden to the mantis by a cardboard barrier) on which a black dot appears when the target and distractor are displayed.

Journal of Experimental Biology • Supplementary information

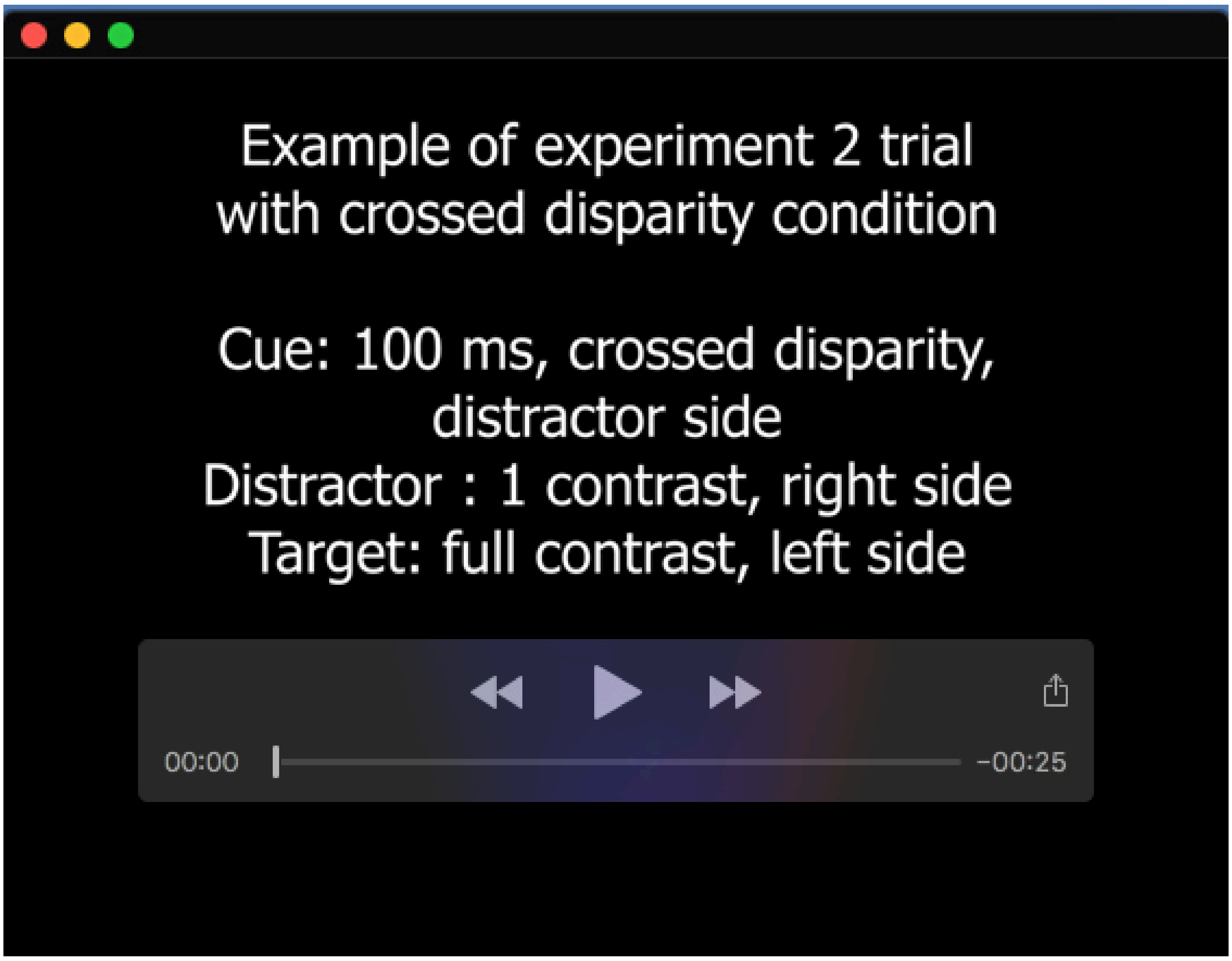

Movie 2. Examples of trials presented to mantises during the experiment 2.
This experiment used 100 ms cues. The first trial presents a crossed disparity cue on the side of a full contrast the distractor. The second trial shows an uncrossed disparity cue presented on the target side and with a 0.25 contrast distractor. The third trial shows a Zero disparity cue placed on the distractor side, with a 0.49 contrast distractor. The fourth trial is Uncued with a 0.25 distractor. Please see additional remarks in the movie 1 caption.

Journal of Experimental Biology • Supplementary information

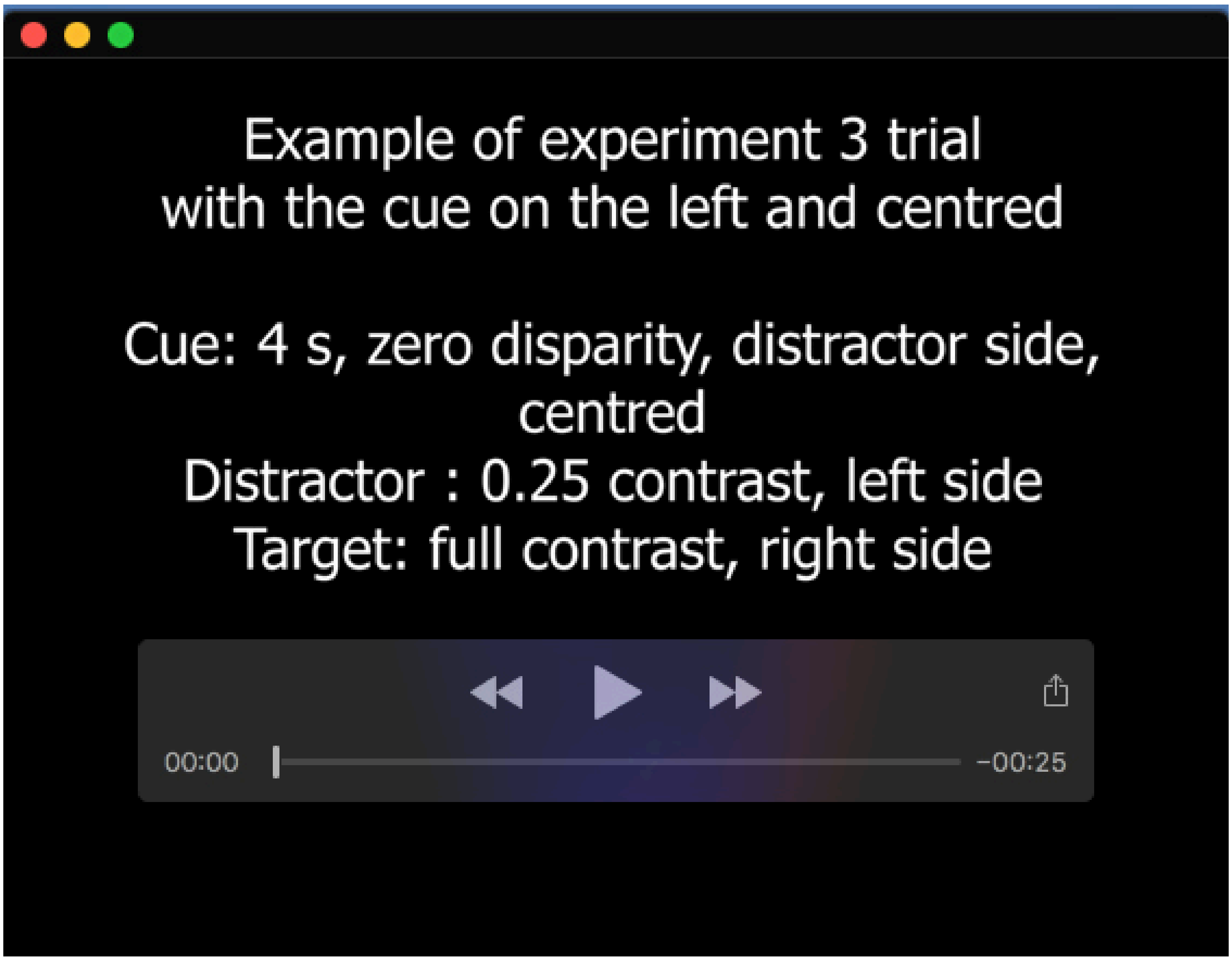

Movie 3. Examples of trials presented to mantises during the experiment 3. In thisexperiment, the cue lasted 4 s and was always presented with a Zero disparity. Compared to the experiment 1 and 2, the cue could either be shifted towards the centre of the screen (centred) or towards the edge of the screen (excentred). The first trial has the cue centred and shown on the side of a 0.25 contrast distractor. The second trial also presents the cue on the side of the distractor but with the excentred condition and 0.75 distractor contrast. The third trial shows an excentred cue placed on the target side and with a 0 contrast distractor (invisible). Finally, the fourth trial shows a centred cue placed on the target side with a 0.49 contrast distractor. Please see additional remarks in the movie 1 caption.
